# Non-Destructive and Real-Time Discrimination of Normal and Frozen-Thawed Beef Based on a Novel Deep Learning Model

**DOI:** 10.3390/foods14193344

**Published:** 2025-09-26

**Authors:** Rui Xi, Xiangyu Lyu, Jun Yang, Ping Lu, Xinxin Duan, David L. Hopkins, Yimin Zhang

**Affiliations:** 1 Shandong Key Laboratory of Intelligent Production Technology and Equipment for Facility Horticulture, Shandong Engineering Research Center of Agricultural Equipment Intelligentization, Shandong Agricultural University, Tai’an 271018, China; xirui@sdau.edu.cn (R.X.);; 2 Lab of Beef Processing and Quality Control, Shandong Agricultural University, Tai’an 271018, China

**Keywords:** YOLO-NF model, frozen-thawed beef, deep learning, model decision, food safety

## Abstract

Discrimination between normal (fresh/non-frozen) and frozen-thawed beef is crucial for ensuring food safety. This paper proposed a novel, non-destructive and real-time you only look once for normal and frozen-thawed beef discrimination (YOLO-NF) model using deep learning techniques. The simple, parameter-free attention module (SimAM) and the squeeze and excitation (SE) attention mechanism were introduced to enhance the model’s performance. A total of 1200 beef samples were used, with their images captured by a charge-coupled device (CCD) camera. In the model development, specifically, the training set comprised 3888 images after data augmentation, while the validation set and test set each included 216 original images. Experimental results on the test set showed that the YOLO-NF model achieved precision, recall, F1-Score and mean average precision (mAP) of 95.5%, 95.2%, 95.3% and 98.6%, respectively, significantly outperforming YOLOv7, YOLOv5 and YOLOv8 models. Additionally, gradient-weighted class activation mapping (Grad-CAM) was adopted to interpret the model’s decision basis. Moreover, the model was deployed on the web interface for user convenience, and the discrimination time on the local server was 0.94 s per image, satisfying the requirements for real-time processing. This study provides a promising technique for high-performance and rapid meat quality assessment in food safety monitoring systems.

## 1. Introduction

Beef is an important part of the global meat industry and is favored by most consumers for its high nutritional value. In recent years, per capita beef consumption and total beef consumption have increased dramatically due to population growth and increased incomes [1]. Freezing can effectively preserve the nutrients in beef, however, the ice crystals formed during the freezing process can cause physical and chemical damage to the meat, resulting in a loss of quality and sensory characteristics [2]. Despite these quality differences, it is hard for consumers to directly distinguish between frozen-thawed and normal (fresh/non-frozen) beef according to the appearance. Mislabelling for normal and frozen-thawed beef can occur both intentionally and unintentionally, which seriously breaches consumers rights and interests, and may also lead to food safety issues. Indeed, discrimination between normal and frozen-thawed beef has long been a necessity in the food industry to guarantee consumers assurance in terms of both economic and food quality aspects [3,4].

Currently, the leading non-invasive technologies used for assessing meat quality include spectroscopic technologies, ultrasonic technology, magnetic resonance imaging (MRI) and nuclear magnetic resonance (NMR) technology [5,6,7,8,9,10], as well as deep learning technologies [11,12]. Spectral information of the object to be measured can be obtained by spectroscopic technologies and non-invasive detection can be achieved. Yu et al. [13] studied the discrimination of fresh, frozen-stored and frozen-thawed beef cuts utilizing hyperspectral imaging technology and achieved accuracies of 92.8% using partial least squares discriminant analysis (PLS-DA), 97.8% using support vector machine (SVM) and 95.0% using back propagation artificial neural network (BP-ANN). Ultrasonic technology relies on two basic parameters, ultrasonic signal and ultrasonic velocity, to assess beef characteristics. Wu et al. [14] evaluated the effects of ultrasonic-assisted thawing at different frequency modes on the thawing efficiency and quality evaluation of frozen beef, and the results showed that ultrasound-assisted thawing treatments with single-frequency and dual-frequency have potential application in detecting thawed beef meat. MRI and NMR both have high sensitivity for distinguishing fresh and frozen-thawed beef, as the strength of their magnetic fields depends on the presence of magnetic atoms in the samples. Perez-Palacios et al. [15] evaluated the capability of MRI and computer vision techniques to classify fresh and frozen-thawed beef and predict physico-chemical, texture and sensory characteristics, and the results revealed that the computational analysis of MRI, especially the algorithm to analyze the image, may be set as a function of the aim and of the type of sample, while the analyzed characteristic was not relevant.

These studies have demonstrated the significance of the abovementioned technologies in assessing normal and frozen-thawed beef. However, some challenges such as high costs, complex technological requirements and data processing, and difficulty to achieve real-time applications persist during implementation. For example, redundant or irrelevant spectral features may be present due to the high dimensionality of spectral data, resulting in models that perform well on training data but poorly on test data, thus spectral feature selection and optimization need to be completed before modeling. Specialized equipment is required for the use of ultrasonic technology and MRI and NMR technology, which is costly. Besides, specialized technicians are needed for operation and data analysis. Therefore, a non-destructive and real-time assessment method is urgently needed to achieve discrimination between normal and frozen-thawed beef.

Deep learning excels in automatic feature extraction, which can automatically extract and learn useful features from raw data without human intervention, thereby reducing human bias while avoiding intricate feature engineering [16,17]. Given the advantages highlighted above, deep learning has been widely employed in meat quality assessment [18,19,20,21]. For example, Abd Elfattah et al. [22] assessed meat freshness using an optimized deep learning method, and achieved an accuracy of 98.5%, sensitivity of 98.5%, and specificity of 99.2%. Lin et al. [23] used fluorescent sensing array and deep learning to monitor meat (beef, chicken and pork) freshness. SqueezeNet, a lightweight convolutional neural network, was applied to automatically identify the freshness level of the meats based on fluorescent sensor array images, obtaining an accuracy of 98.2%. Huang et al. [24] used an improved ResNet-50 model to achieve automatic recognition and classification of pork primal cuts, and the identification accuracy reached 94.5%.

However, to the authors’ knowledge, research into the discrimination of normal and frozen-thawed beef based on deep learning is scarce. In this paper, a novel you only look once for normal and frozen-thawed beef (YOLO-NF) model was proposed and deployed utilizing deep learning techniques to achieve non-destructive and real-time discrimination of normal and frozen-thawed beef. The simple, parameter-free attention module (SimAM) and the squeeze and excitation (SE) attention mechanism were adopted in the YOLO-NF model to enhance the discrimination performance of the model. Moreover, the proposed model was deployed on the web interface to improve the accessibility for users. It is hoped that the current research should provide an accurate, non-destructive and real-time detection method for identifying normal and frozen-thawed beef.

## 2. Materials and Methods

**Figure 1:**
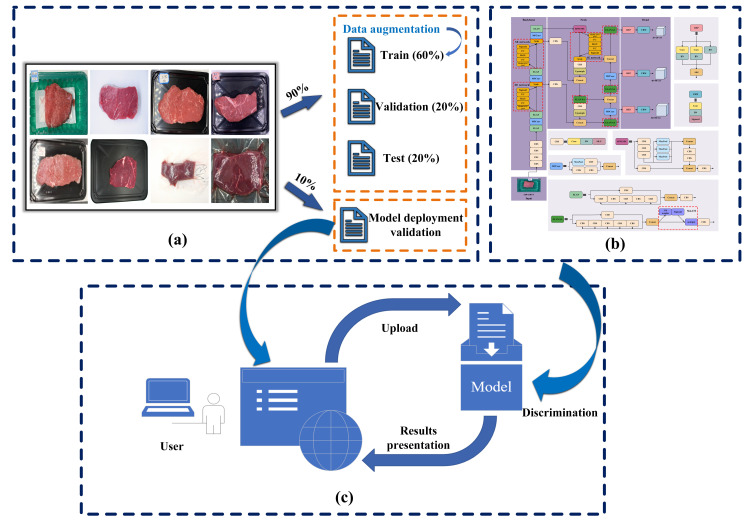
The workflow of the discrimination method in this study. (**a**) Dataset preparation. For sample images, first row: normal beef; second row: frozen-thawed beef. (**b**) Discrimination model. (**c**) Model deployment.

A flowchart of the proposed method for discrimination of normal and frozen-thawed beef is shown in Figure 1. The pipeline workflow consists of data preparation, a novel model development and deployment of the discrimination model. Data augmentation methods and deep learning techniques were used in the process of data pre-processing and development of the discrimination model, respectively, and the model deployment was carried out based on the Gradio deep learning framework.

### 2.1. Data Acquisition and Pre-Processing

**Figure 2:**
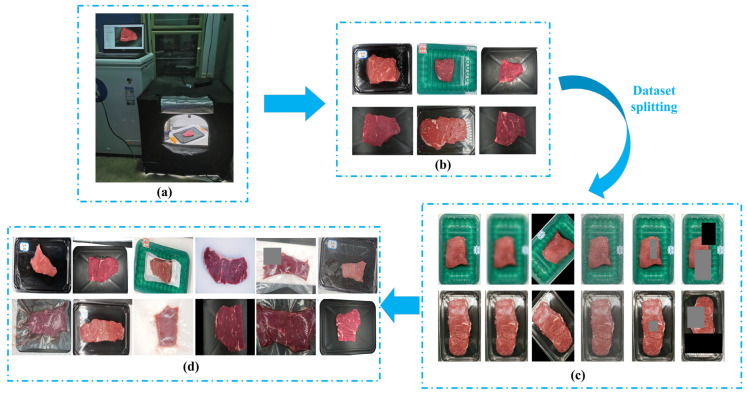
Flowchart of data acquisition and pre-processing. (**a**) Data acquisition equipment. (**b**) Partial beef sample images. First row: normal beef; second row: frozen-thawed beef. (**c**) Data augmentation for training set. First row: normal beef; second row: frozen-thawed beef. (**d**) Partial images for model training. First row: normal beef; second row: frozen-thawed beef.

As shown in Figure 2a, the image acquisition equipment consisted of a charge-coupled device (CCD) camera (LBAS-GE60-17M/C, LUSTER LightTech Co., LTD. (Luster), Beijing, China) with a pixel size of 3072 × 2048, a circular light source, a collection shed (0.6 m × 0.6 m × 0.6 m) and a personal computer. All samples in this study were from our previous work [3,25,26]. Briefly, the *M. longissimus lumborum* were obtained at 24 h postmortem from both sides of 114 Simmental crossbred bulls (Age: 18–24 months), and the loins were cut into 2.54~3 cm thick steaks in a commercial beef harvest facility. All steaks were vacuum-packed and transported to the laboratory in ice within 3 h. A total of 1200 samples were used in this study, including 700 normal samples and 500 frozen-thawed samples. Some of the sample images are shown in Figure 2b. 90% of the sample images (1080 images, normal: 630, frozen-thawed: 450) were used for model development, and the remaining 10% (120 images, normal: 70, frozen-thawed: 50) were used for model deployment validation. The 1080 images were randomly split into training, validation and test sets at a 6:2:2 ratio (648, 216 and 216 images, respectively) for model development, with the normal-to-frozen-thawed ratio consistently maintained at 7:5 across all subsets. To enhance sample diversity and model robustness, data augmentation techniques (blurring, rotation, additive Gaussian noise, random erasing and rand-augment, see Figure 2c) were applied to the training set. This process generated 3240 new images, which were combined with the original 648 training images, yielding a total of 3888 images (normal: 2268, frozen-thawed: 1620) for model training.

### 2.2. The Proposed YOLO-NF Model

#### 2.2.1. Overall Network Structure of the YOLO-NF Model

**Figure 3:**
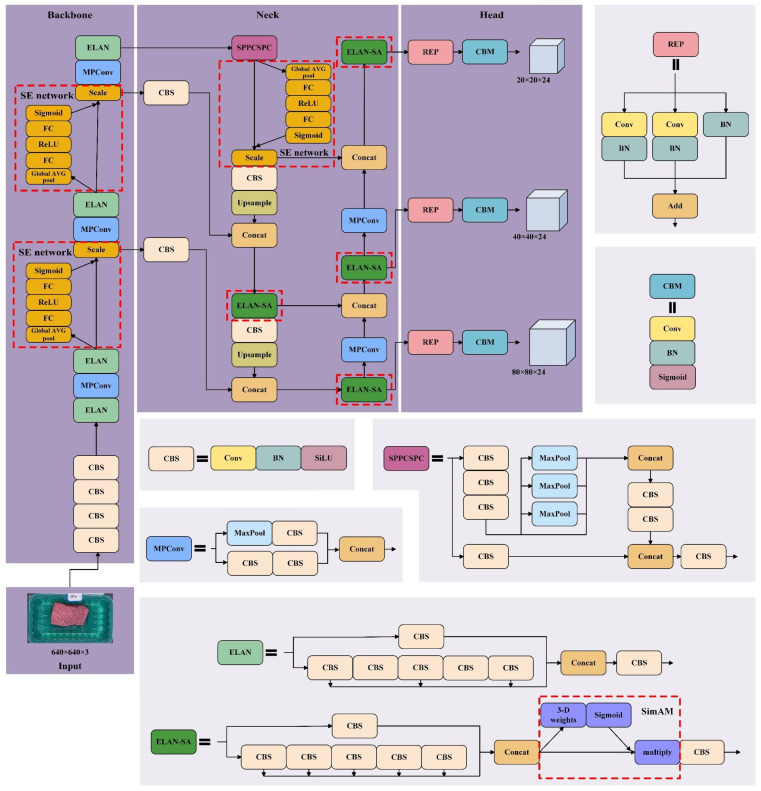
Overall network structure of the YOLO-NF model. Within this structural diagram, dark backgrounds denote the network structure sections, light-colored filled regions provide supplementary explanations of modules within these sections, and core improved modules are distinctly highlighted using red dashed boxes.

To achieve non-destructive and real-time discrimination for normal and frozen-thawed beef, a novel YOLO-NF model was proposed in this study. Based on the YOLOv7 model [27,28], the SimAM module and the SE attention mechanism module were integrated into the neck and backbone networks to enhance the model’s abilities of feature extraction and global information perception, thus improving the discrimination performance of the model. The overall network structure of the YOLO-NF model is shown in Figure 3.

#### 2.2.2. Network Structure of YOLOv7 Model

YOLO, a real-time object detection algorithm, transforms the object detection task into a regression problem, enabling the prediction of object bounding boxes and class probabilities via a single neural network in one forward pass, which is suitable for scenarios demanding real-time processing. The YOLOv7 model is part of the successive versions developed and improved based on the original YOLO object detection model. It has inherited the basic ideas of the YOLO model while introducing distinct optimizations and enhancements.

**Figure 4:**
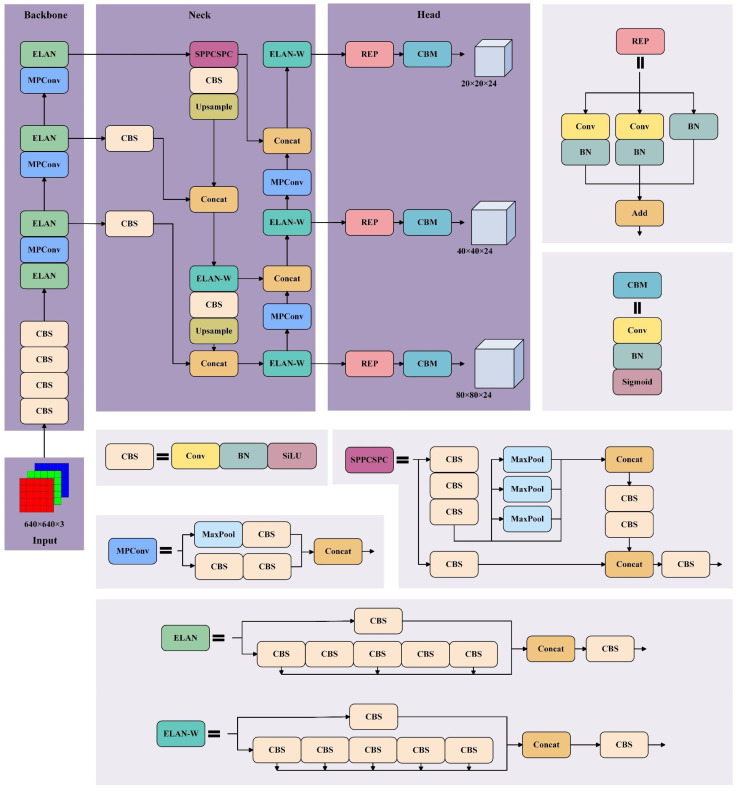
Network structure of the original YOLOv7 model. Within this structural diagram, dark backgrounds denote the network structure sections, and light-colored filled areas offer supplementary descriptions of modules within these sections.

The YOLOv7 model (baseline model) is composed of four main parts: Input, Backbone, Neck and Head, as shown in Figure 4. The input module scales the input image to a unified pixel size to reduce the computation cost. The backbone network comprises the Conv-BN-SiLU (CBS) module for feature extraction, the efficient layer aggregation network (ELAN) module for accelerating model convergence by combining CBS through multiple branches, and a maxpooling and convolution (MPConv) module for improving the feature extraction ability. The neck network employs CBS, ELAN-W, MPConv and spatial pyramid pooling cross-stage partial convolution (SPPCSPC) modules to refine and enhance the features extracted from the backbone layers by feature fusion. The ELAN-W module differs from ELAN only in the concatenate operation. The SPPCSPC module is used to change the channel dimensions, which consists of max pooling and CBS module.

The head network consists of the reparameterization (REP) module and the Conv-BN-Sigmoid (CBM) module. The REP module was used to adjust three different scale outputs from neck layers by combining the convolution (Conv) layers and batch normalization (BN) layers. The CBM module is used to predict the targets and create anchor frames.

#### 2.2.3. SimAM Module

**Figure 5:**
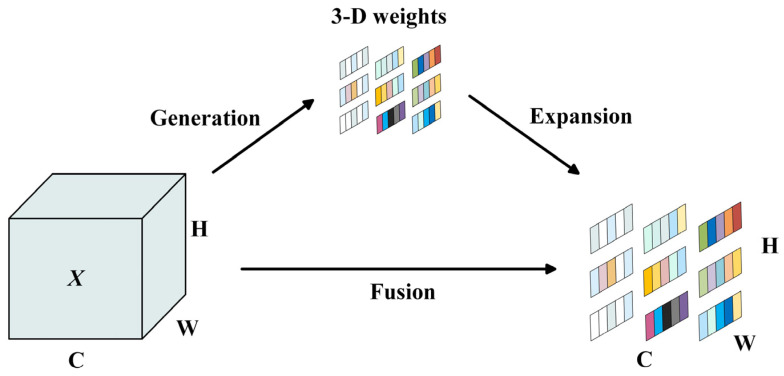
Schematic diagram of the SimAM module.

The SimAM module [29] could adaptively adjust the weight of each pixel by calculating the similarity between each pixel in the feature map and its surrounding pixels and infer 3-D attention weights for the feature map in a layer without adding parameters to the original networks, thus enhancing representation of feature maps and improving model performance. This well-established attention mechanism has been widely validated and applied in deep learning. The schematic diagram of the SimAM module is shown in Figure 5.

Aiming at achieving better weights allocation, the SimAM module constructs an energy function based on neuroscience theory to find the important neural by measuring the linear differentiability between neurons. The function is defined as follows.

(1) $$e_{t}^{*}=\frac{4\left(\overset{\wedge 2}{\sigma }+\lambda \right)}{{\left(t-\overset{\wedge }{\mu }\right)}^{2}+2\overset{\wedge 2}{\sigma }+2\lambda }$$

where $e_{t}^{*}$ stands neuron importance, *t* is the target neuron of the input feature in the current channel, $\overset{\wedge }{\mu }$ and $\overset{\wedge 2}{\sigma }$ are the mean and variance of all neurons in the channel to avoid repeated calculations and reduce the computational costs, and λ represents the weight constant. Equation (1) indicates that the lower $e_{t}^{*}$, the greater the difference between neuron t and its surrounding neurons, and the higher its importance. Finally, excessive values are suppressed by Sigmoid function and the formula is as follows.

(2) $$\tilde{X}=Sigmoid\left(\frac{1}{e_{t}^{*}}\right)⊙X$$

**Figure 6:**
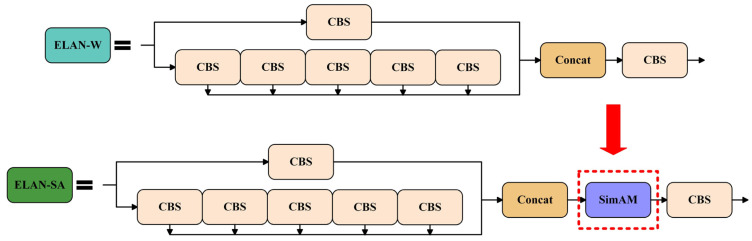
Schematic diagram of the ELAN-SA module.

In order to enhance the feature extraction capability of the model, the SimAM module was integrated into YOLOv7 network. As shown in Figure 6, we proposed the ELAN-SA network by adding SimAM after the concatenation layer of the ELAN-W module, which adaptively emphasizes critical pixel-level features.

The ELAN-SA module inherits the existing features of ELAN-W module and it allows for more focused processing of sample features. The ELAN-SA module was added before the concatenate layers and output layers of the neck network to enhance the feature refinement ability and feature fusion efficiency of model.

#### 2.2.4. SE Attention Mechanism Module

**Figure 7:**
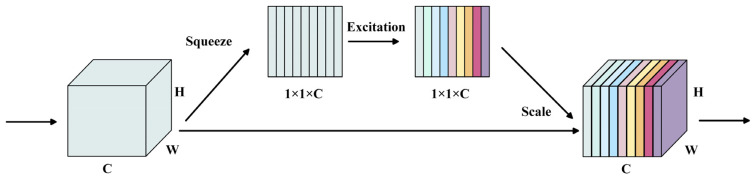
Schematic diagram of the SE network.

The SE attention mechanism [30] compresses the spatial dimensions of the feature maps through global average pooling operations, which makes global context information available for each channel and enhances the capability to perceive global features of the model. It has been widely applied in deep learning frameworks. As shown in Figure 7, the SE network consists of three main steps: Squeeze, Excitation and Scale operation.

The squeeze operation could implement image compression by global average pooling, the squeeze operation equation for the channel is shown as follows.

(3) $$z_{c}=F_{sq}\left(u_{c}\right)=\frac{1}{H\times W}\sum_{i=1}^{H}\sum_{j=1}^{W}u_{c}\left(i,j\right),z\in R^{c}$$

where $u_{c}\left(i,j\right)$ is the value of each point on the feature map channel.

The excitation operation could obtain a weighting coefficient for the importance of each channel by using two fully connected layers. The first fully connected layer is used to reduce the channel dimension and the other is used to restore the channel dimension. In the process, ReLU activation function is used for nonlinear transformation. Finally, weighting coefficients are normalized by Sigmoid activation function.

The scale operation could realize the assignment of channel importance by multiplying the normalized weights coefficients $s_{c}$ obtained previously for each channel with the original eigenvalue $u_{c}$, which is presented in Equation (4).

(4) $$F_{scale}\left(s_{c},u_{c}\right)={\tilde{x}}_{c}=s_{c}\times u_{c}$$

**Figure 8:**
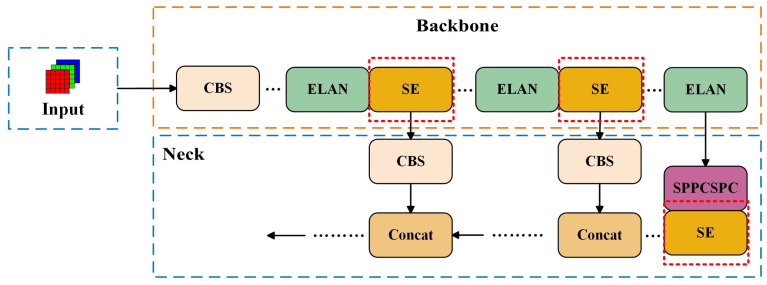
Network structure with the SE attention mechanism.

To enhance the capability to focus on beef features of the model, the SE attention mechanism was adapted in this study. As shown in Figure 8, the SE attention mechanism was inserted between the backbone and neck networks. By weighting the beef feature maps from the backbone, SE highlights prominent features, enabling the neck network to fully fuse discriminative information for accurate differentiation.

### 2.3. Evaluation Metrics

Some evaluation metrics such as precision (P), recall (R), F1-Score, mean average precision (mAP) were introduced to evaluate the performance of the beef discrimination model. The above metrics are as follows.

(5) $$Precision=\frac{TP}{TP+FP}$$

(6) $$Recall=\frac{TP}{TP+FN}$$

(7) $$F1-Score=2\times \frac{P\times R}{P+R}$$

(8) $$mAP=\frac{1}{n}\sum_{i=0}^{n}AP_{i}$$

where *TP* is true positive, *TN* is true negative, *FP* represents false positive and *FN* represents false negative.

The precision indicator measures the proportion of true positive samples in the predicted positive samples. The recall indictor presents the proportion of correctly predicted positive samples to the total positive samples. F1-Score is the harmonic mean of precision and recall and provides a balance between the two.

The Precision-Recall curve can be achieved by considering Recall and Precision as the *x*-axis and *y*-axis, respectively, and the area under the curve represents the value of average precision (AP). The mean average precision (mAP) is the average value of AP, which is a crucial indicator to evaluate the performance of the beef discrimination model, and the higher the value is, the better the discrimination effect of the model. The commonly used indicator about the mAP is mAP@0.5, which is often employed in the Precision-Recall curve and represents the average precision of AP for an intersection over union (IoU) of 0.5.

### 2.4. Supplementary Model Analyses

Qualitative analysis, ablation experiments, and model interpretation are supplementary to evaluation metrics.

Qualitative analysis visually presents misidentified samples to intuitively explain model behavior, compensating for quantitative metric limitations. Ablation experiments validate module effectiveness via four models (baseline, baseline + SimAM, baseline + SE, YOLO-NF) under consistent parameters. Model interpretation uses convolutional layer feature map extraction and activation heatmaps generated by Gradient-Weighted Class Activation Mapping (Grad-CAM) to reveal decision bases and enhance interpretability.

### 2.5. Model Deployment

**Figure 9:**
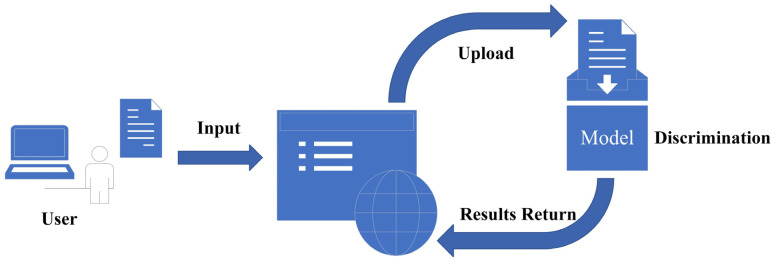
Flowchart of the model deployment.

For user-friendliness, the proposed model was deployed on the web interface based on the Gradio framework. Gradio is a python-based framework for deep learning applications, and the user interface built with it can be shared with others through web connections. The flowchart of the model deployment is composed of the front-end and the back-end, as depicted in Figure 9. Users upload the beef images required to be identified through the front-end interface. Subsequently, the discrimination task is performed by the back-end after receiving these uploaded images. Then, the results are presented to the front-end interface.

## 3. Results and Discussion

### 3.1. Experimental Setup

All experiments were carried out on a personal computer running Python 3.8 and Pytorch 2.3.0, equipped with Intel (R) Core (TM) i9-14900HX CPU, NVIDIA GeForce RTX 4060 GPU and CUDA 11.8. The SGD optimizer with CIoU loss function was employed in this study. The YOLO-NF model was trained over 80 epochs on the above personal computer with an initial learning rate of 0.0001 and a final learning rate of 0.1, more hyperparameter configurations are listed in Table 1.

### 3.2. Experimental Training Process

**Figure 10:**
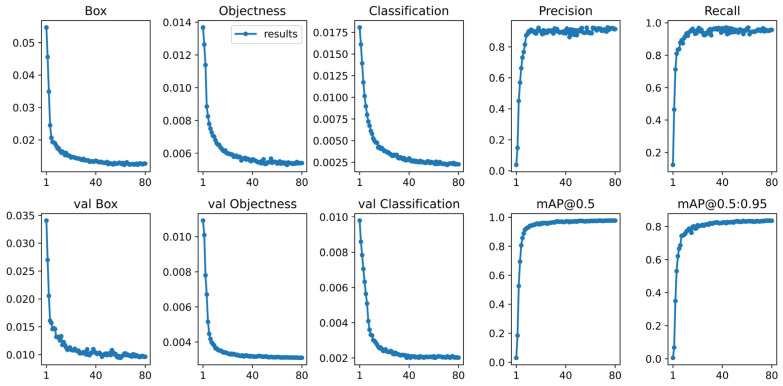
Model training and validation results. First row: results on the training set; second row: results on the validation set.

All images were scaled to 640 × 640 pixels size to reduce the computation effort in the training process. The training process are displayed in Figure 10. The values of the overall loss exhibited a decreasing trend and reached convergence after 80 epochs. The precision and recall increased gradually with the increase of epochs and reached a peak value sta-bilizing around it after 80 epochs. These results indicate that the proposed YOLO-NF model has strong fitting and generalization ability and is suitable for identification of normal and frozen-thawed beef.

### 3.3. Quantitative Analysis

Experimental results on the test set indicated that the YOLO-NF model achieved a precision of 95.5%, a recall of 95.2%, and thus obtained an F1-Score of 95.3%. The mAP was 98.6%, and the average running time per image was 0.022 s, meeting the demand for real-time identification [31].

To validate the performance of the proposed model for normal and frozen-thawed beef discrimination, quantitative comparisons were conducted with YOLOv7, YOLOv8 and YOLOv5 models [32]. These models were retrained in this study using the same dataset and experimental setup as the proposed YOLO-NF model, with consistent experimental setup, initial training parameters, dataset, and evaluation metrics across all mentioned models. The statistical results in Table 2 show that the proposed model has the best discrimination performance, followed by the YOLOv7 model, then YOLOv8 and YOLOv5 models.

Precision of the YOLO-NF model was 12.7%, 21.9% and 16.2% higher than that of YOLOv7, YOLOv8 and YOLOv5 models, respectively. Recall of the YOLO-NF model was improved by 15.5%, 10.6% and 11.9%, respectively, compared with YOLOv7, YOLOv8 and YOLOv5 models. The F1-Score of the YOLO-NF model was 14.1%, 16.6% and 14.1% higher than that of the above models, respectively. The mAP of the YOLO-NF model was 8.7%, 13.6% and 13.8% higher than that of the above models, respectively. The average running time per image of the YOLO-NF model was 0.022 s, higher than that of YOLOv7, YOLOv8 and YOLOv5 models. This suggests a general problem in deep learning that improving model performance usually requires an increase in model complexity, that is, it increases the floating point operations (FLOPs), which can reduce the model efficiency [33].

Discrimination performance indicators of the proposed model were all improved compared with the baseline model, and this indicates that the SimAM and SE modules can significantly improve the discrimination performance of the model. Meanwhile, the addition of these modules increases the model complexity, which in turn leads to longer running times. This is consistent with the common trade-off between performance and efficiency in deep learning as mentioned earlier.

**Figure 11:**
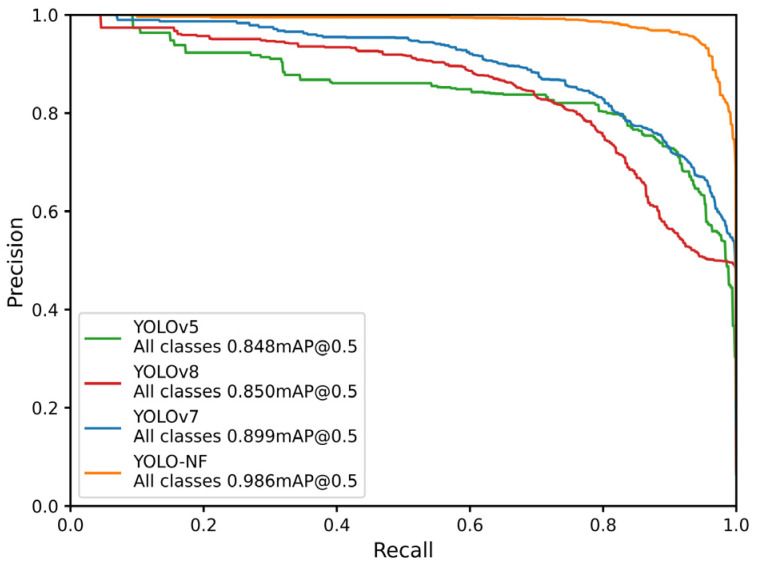
Precision-Recall curves of discrimination results for all classes with the proposed YOLO-NF model and other models (YOLOv5, YOLOv7 and YOLOv8).

Precision-Recall curves of discrimination results for all classes of the above models are shown in Figure 11. The overall discrimination performance of the YOLO-NF model was significantly better than other models, followed by YOLOv7 model.

**Figure 12:**
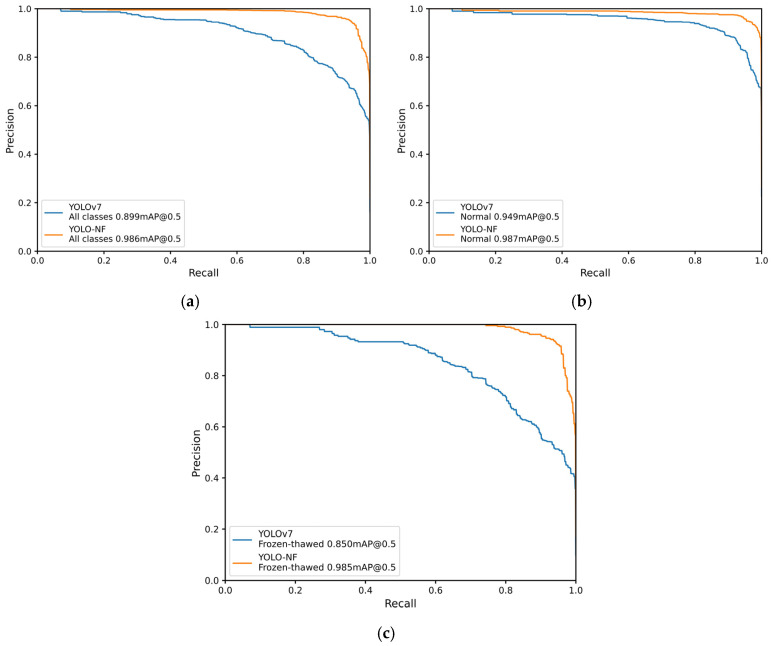
Precision-Recall curves of the proposed YOLO-NF model and YOLOv7 model. (**a**) Precision-Recall curves for all classes discrimination. (**b**) Precision-Recall curves for normal beef discrimination. (**c**) Precision-Recall curves for frozen-thawed beef discrimination.

To further explore the discrimination results of the YOLO-NF model compared with the baseline model, Precision-Recall curves of the YOLO-NF model and YOLOv7 model for all classes, normal beef, frozen-thawed beef discriminations are shown in Figure 12, respectively. The overall mAP for beef discrimination of the YOLO-NF model was 98.6%, and outperformed the 89.9% achieved by YOLOv7 model. The mAPs for normal and frozen-thawed beef discrimination of the YOLO-NF model were 98.7% and 98.5%, respectively, which were 3.8% and 13.5% higher than those of YOLOv7 model, respectively.

**Figure 13:**
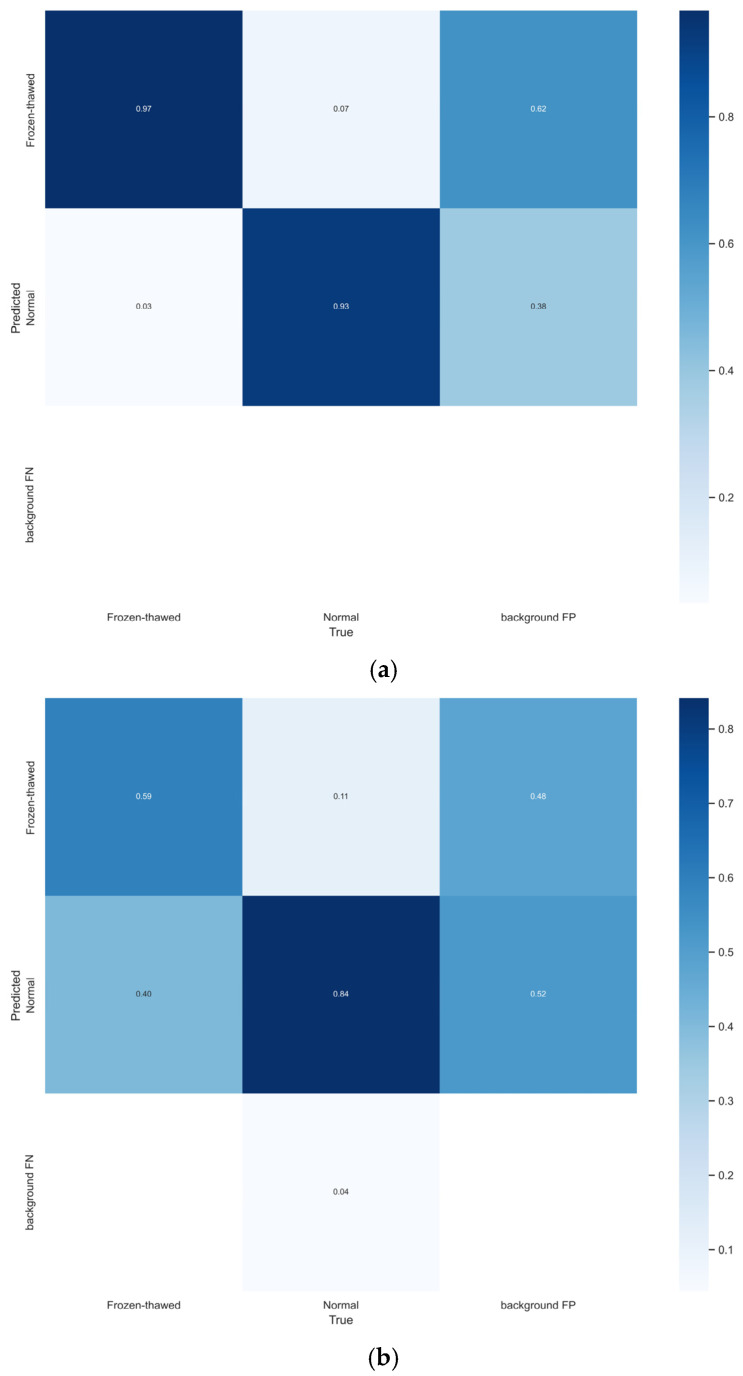
Confusion matrices for discrimination of normal and frozen-thawed beef. (**a**) The proposed YOLO-NF model. (**b**) The baseline (YOLO v7) model.

Table 3 shows more detailed quantitative comparisons. And Figure 13 displays the confusion matrices of the YOLO-NF model and YOLOv7 model for normal and frozen-thawed beef discrimination, which all demonstrate that the proposed YOLO-NF model has achieved strong discrimination performance, especially on frozen-thawed beef.

### 3.4. Qualitative Analysis

**Figure 14:**
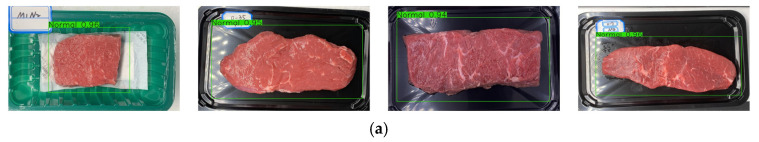

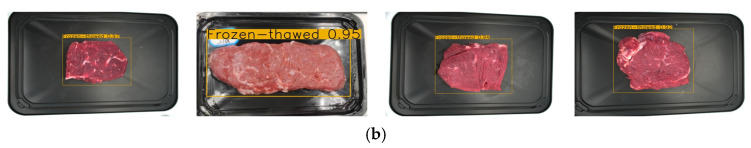
Some discrimination results of the YOLO-NF model. (**a**) Discrimination results of normal beef. (**b**) Discrimination results of frozen-thawed beef.

Qualitative discrimination results for normal and frozen-thawed beef of the YOLO-NF model are shown in Figure 14. As can be seen from it, the YOLO-NF model can achieve accurate discrimination for normal and frozen-thawed beef.

**Figure 15:**
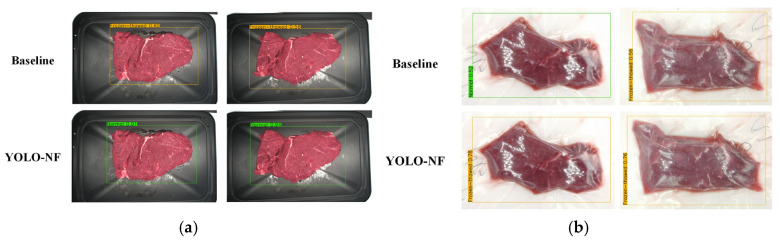
Comparison of qualitative discrimination results between the YOLO-NF model and baseline (YOLOv7) model. (**a**) Normal beef. (**b**) Frozen-thawed beef.

Qualitative comparisons of discrimination results between the YOLO-NF model and the baseline model and were also investigated, as presented in Figure 15. Discrimination results for normal beef of the YOLO-NF model and the baseline model are displayed in Figure 15a, from which the normal beef samples were misidentified as the frozen-thawed beef by the baseline model, whereas the above samples were correctly identified by the YOLO-NF model. Discrimination results of the YOLO-NF model and baseline model for frozen-thawed beef are shown in Figure 15b, where one frozen-thawed beef sample was mistakenly detected as normal beef by the baseline model, whereas it was correctly categorized by the YOLO-NF model, and the other frozen-thawed beef sample was correctly discriminated by the baseline model, but its confidence was much lower than that of the YOLO-NF model. The qualitative analysis indicates that the SimAM and SE modules in the YOLO-NF model can make the model focus more on sample features while enhancing its ability to perceive global information, thereby improving the model’s discrimination performance.

### 3.5. Ablation Experiments

A series of ablation experiments were carried out to analyze the performance enhancement brought by different modules. The performance comparison results with different schemes are presented in Table 4. Scheme A was the baseline model. Scheme B was the baseline model with the SimAM module, which could dynamically adjust the weight of each pixel to enhance important features and suppress irrelevant ones. Scheme C presented the baseline model with the SE module, which could enhance the perception ability of the model for global features. Scheme D indicated the YOLO-NF model, which utilized the SimAM and SE modules to improve the performance for discrimination of normal and frozen-thawed beef.

Scheme B outperformed Scheme A with an improvement of 11.6% in precision, 9% in recall, 10.3% in F1-Score and 6.9% in mAP. This indicates that the SimAM module can focus more on target features and suppress irrelevant information, thus improving the model performance.

Scheme C was superior to Scheme A in discrimination performance, especially in the indicator of recall, which was improved by 14.5%, and the other indicators were improved by 4.8% to 10.6%. This showed that the SE module made global context information available for each channel and enhanced the ability of the model to perceive global features.

Compared with Scheme A, the model performance was significantly enhanced in Scheme D (P = 95.5%, R = 95.2%, F1-Score = 95.3%, mAP = 98.6%), precision increased by 12.7%, recall increased by 15.5%, F1-Score increased by 14.1% and mAP increased by 8.7%, respectively. By incorporating the SimAM and SE modules, Scheme D can significantly improve the feature extraction and localization capabilities of the model, thereby improving the precision and recall, and ultimately enhancing the model performance.

### 3.6. Model Interpretations

**Figure 16:**
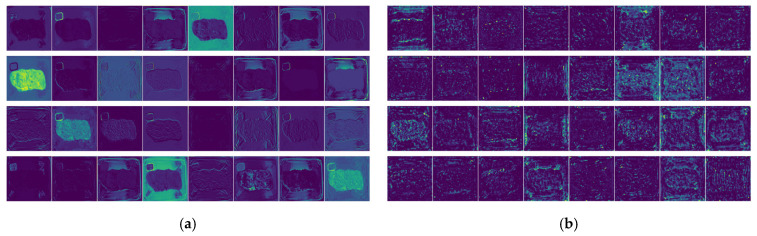

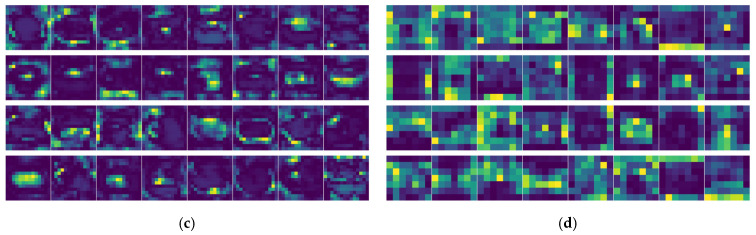
Feature maps visualization. (**a**) Feature maps produced by Layer_0. (**b**) Feature maps produced by Layer_14. (**c**) Feature maps produced by Layer_35. (**d**) Feature maps produced by Layer_90.

Feature maps obtained from different convolutional layers were extracted to interpret the model’s learning process, as shown in Figure 16. Shallow convolutional layers are used to extract the low-level features such as edges, contours, and textures. The feature maps produced by these layers have a high degree of similarity to the original image, with clear details and almost all the information of the original image included, as shown in Figure 16a. As the network goes deeper, the feature maps become increasingly abstract, as shown in Figure 16b,c. In the feature maps output by deep convolutional layers, information about the original image decreases, while that about the abstract semantics of the target increases, as shown in Figure 16d. Through the combined operations of multiple convolutional layers in the model, target features can be effectively extracted.

**Figure 17:**
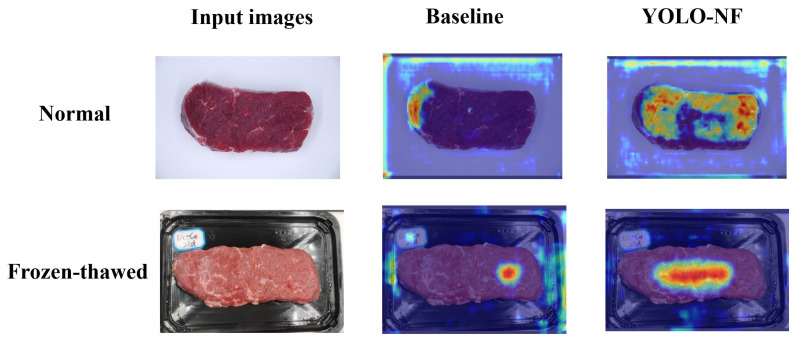
Activation heat maps of the YOLO-NF model and the baseline (YOLOv7) model for normal and frozen-thawed beef discrimination.

The Gradient-Weighted Class Activation Mapping (Grad-CAM) method was utilized to generate the activation heat maps by mapping the category prediction to the corresponding spatial location of the original image. Heat maps provides an intuitive representation of the areas that the model focuses on and its sensitivity to certain features, which could help us to understand the decision basis for the model. The redder the color, the more attention is paid to the area. As shown in Figure 17, less focus was placed on sample features by the baseline model, whereas our proposed YOLO-NF model devoted more attention to such features. It is because the SimAM and SE modules in the YOLO-NF model have enhanced the sample feature weights and the model’s ability to perceive global features. This also explains why the discrimination performance of our proposed YOLO-NF model is better than that of the baseline model.

### 3.7. Deployment of the YOLO-NF Model

**Figure 18:**
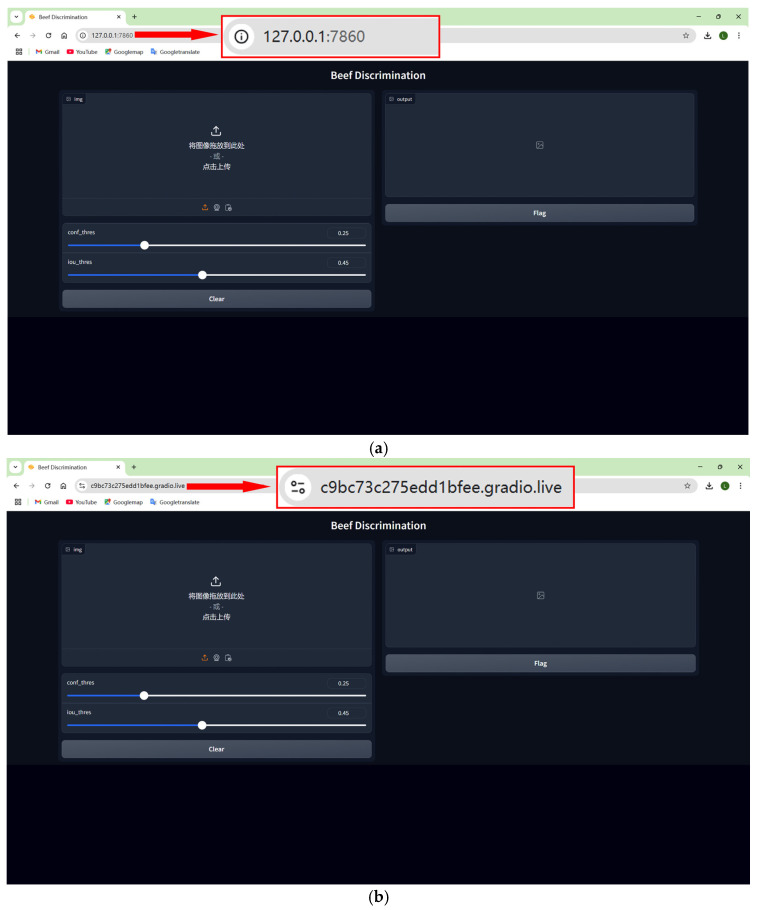
User interfaces of the deployed model under different deployment methods. (**a**) Interface for the locally deployed model. (**b**) Interface for the model deployed via public URL. In the figure, the Chinese text “将图像拖放到此处-或-点击上传” is defined as “Drag and drop the image here-or-click to upload”, which instructs users on how to upload an image for beef discrimination.

The Gradio framework was adopted in the deployment of the proposed model, and the user interface built with it can be shared with others through web connections. If the share parameter is set to “False”, it means that the deployed model is only available on the local server and can only be accessed by a few people, as shown in Figure 18a, which enhances the security of the data. If the share parameter is set to “True”, a public uniform resource locator (URL) will be created, as shown in Figure 18b, which enables the deployed model to be accessed through a browser, thus improving the accessibility for users.

**Figure 19:**
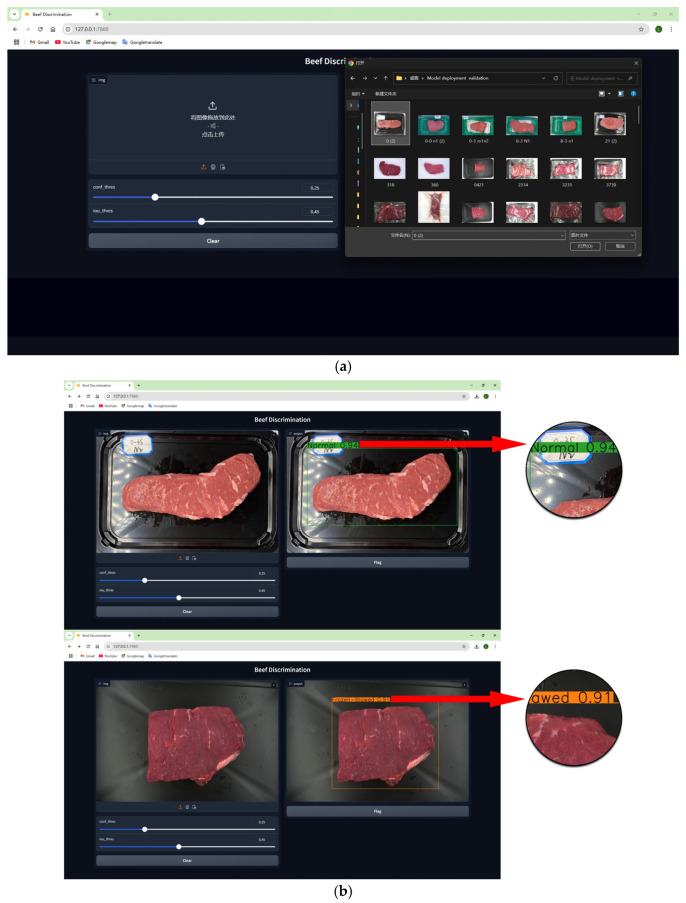
Local deployment discrimination process. (**a**) Image upload. (**b**) Result presentation to users. In the figure, the Chinese text “将图像拖放到此处-或-点击上传” is defined as “Drag and drop the image here-or-click to upload”, which instructs users on how to upload an image for beef discrimination.

Taking the model deployment on the local server as an example, Figure 19 illustrates the discrimination process in detail. Users could either click the camera icon button to take a picture or upload an image from the photo album to the backend for identification, as shown in Figure 19a, and the identification results are exhibited to users, as depicted in Figure 19b. From uploading images to results return, the process takes about 0.94 s, meeting the demand for real-time identification [31]. Compared to the average running time of 0.022 s on the test set, the increased time is due to data transmission between the front-end and the back-end.

## 4. Conclusions

This study developed and deployed a novel YOLO-NF model to achieve non-destructive and real-time discrimination between normal and frozen-thawed beef using deep learning techniques. Quantitative analyses confirmed that the model achieved a discrimination mAP of 98.6% with an average running time of 0.022 s per image. Ablation experiments and activation heat maps validated the effectiveness of SimAM and SE modules in enhancing feature extraction and clarifying the decision-making basis, respectively. Additionally, deployment via Gradio yielded a total discrimination time of 0.94 s per image on a local server, highlighting its potential for user-friendly on-site use.

These findings advance food quality assessment by providing a rapid, high-performance, and non-invasive approach for beef quality inspection. The high mAP ensures reliable differentiation between normal and frozen-thawed beef, which is pivotal for preventing mislabeling and safeguarding consumer rights. The real-time capability of the model enables its seamless integration into industrial workflows, mitigating reliance on labor-intensive manual inspection and facilitating efficient market supervision.

However, this study has limitations that should be addressed in future work. First, the model was trained and tested on a dataset of 1200 beef samples, which may restrict its generalizability to larger or more diverse populations. Second, validation was limited to beef (*M. longissimus lumborum*), and adaptation to other meat types (e.g., pork, lamb) will require targeted training, though transfer learning could accelerate this process. Third, while an independent dataset (120 samples) was used for deployment validation, external validation with third-party datasets is needed to further confirm the model’s robustness across different acquisition conditions and laboratories.

This study highlights deep learning’s potential in food safety, laying a theoretical and technical foundation for quality control in the meat industry. Future work will focus on expanding the dataset, testing across multiple meat types, and conducting external validation to enhance the model’s practical utility.

## Figures and Tables

**Table 1:** Hyperparameter configurations for all experiments in this study.

Hyperparameter	Configuration
Epoch	80
Initial learning rate	0.0001
Final learning rate	0.1
Batch size	4
Momentum	0.97
Weight decay	0.0005
Input image size	640 × 640

**Table 2:** Quantitative comparisons of discrimination performance between the proposed YOLO-NF model and other models (YOLOv5, YOLOv7 and YOLOv8). Abbreviations: mAP (mean Average Precision) refers to average of AP (Average Precision), a core indicator for evaluating beef discrimination model performance; FLOPs (Floating Point Operations) refers to measure of model computational complexity.

Model	Precision (%)	Recall (%)	F1-Score (%)	mAP@0.5 (%)	FLOPs (G)	Running Time (s)
YOLOv5	79.3	83.3	81.2	84.8	15.8	0.015
YOLOv8	73.6	84.6	78.7	85.0	28.4	0.011
YOLOv7	82.8	79.7	81.2	89.9	103.2	0.0214
YOLO-NF	95.5	95.2	95.3	98.6	103.4	0.022

**Table 3:** Detailed discrimination results of the YOLO-NF model and baseline model. Abbreviations: mAP (mean Average Precision) refers to average of AP (Average Precision), a core indicator for evaluating beef discrimination model performance.

Samples	Model	Precision (%)	Recall (%)	F1-Score (%)	mAP@0.5 (%)
All	Baseline	82.8	79.7	81.2	89.9
	YOLO-NF	95.5	95.2	95.3	98.6
Normal	Baseline	94.2	79.3	86.1	94.9
	YOLO-NF	93.1	99.6	96.2	98.7
Frozen-thawed	Baseline	71.4	80.2	75.5	85.0
	YOLO-NF	97.9	90.9	94.3	98.5

**Table 4:** Ablation experiment results.

Scheme	Baseline	SimAM	SE	Precision (%)	Recall (%)	F1-Score (%)	mAP@0.5 (%)
A	√	-	-	82.8	79.7	81.2	89.9
B	√	√	-	94.4	88.7	91.5	96.8
C	√	-	√	87.6	94.2	91.8	97.1
D	√	√	√	95.5	95.2	95.3	98.6

## Data Availability

The original contributions presented in the study are included in the article, further inquiries can be directed to the corresponding author.
